# Improving the tolerability of osimertinib by identifying its toxic limit

**DOI:** 10.1177/17588359221103212

**Published:** 2022-06-03

**Authors:** Bram C. Agema, G. D. Marijn Veerman, Christi M. J. Steendam, Daan A. C. Lanser, Tim Preijers, Cor van der Leest, Birgit C. P. Koch, Anne-Marie C. Dingemans, Ron H. J. Mathijssen, Stijn L. W. Koolen

**Affiliations:** Department of Medical Oncology, Erasmus MC Cancer Institute, Erasmus University Medical Center, Dr. Molewaterplein 40, Rotterdam 3015 GD, The Netherlands Department of Clinical Pharmacy, Erasmus University Medical Center, Rotterdam, The Netherlands; Department of Medical Oncology, Erasmus MC Cancer Institute, Erasmus University Medical Center, Rotterdam, The Netherlands; Department of Pulmonology, Erasmus MC Cancer Institute, Erasmus University Medical Center, Rotterdam, The Netherlands; Department of Pulmonology, Erasmus MC Cancer Institute, Erasmus University Medical Center, Rotterdam, The Netherlands; Department of Pulmonology, Amphia Hospital, Breda, The Netherlands; Department of Medical Oncology, Erasmus MC Cancer Institute, Erasmus University Medical Center, Rotterdam, The Netherlands; Department of Clinical Pharmacy, Erasmus University Medical Center, Rotterdam, The Netherlands; Department of Pulmonology, Amphia Hospital, Breda, The Netherlands; Department of Clinical Pharmacy, Erasmus University Medical Center, Rotterdam, The Netherlands; Department of Pulmonology, Erasmus MC Cancer Institute, Erasmus University Medical Center, Rotterdam, The Netherlands; Department of Medical Oncology, Erasmus MC Cancer Institute, Erasmus University Medical Center, Rotterdam, The Netherlands; Department of Medical Oncology, Erasmus MC Cancer Institute, Erasmus University Medical Center, Rotterdam, The Netherlands Department of Clinical Pharmacy, Erasmus University Medical Center, Rotterdam, The Netherlands

**Keywords:** exposure–efficacy relationship, exposure–toxicity relationship, NSCLC, osimertinib, preventive dose reduction

## Abstract

**Background:**

Osimertinib is the cornerstone in the treatment of epidermal growth factor receptor-mutated non-small cell lung cancer (NSCLC). Nonetheless, ±25% of patients experience severe treatment-related toxicities. Currently, it is impossible to identify patients at risk of severe toxicity beforehand. Therefore, we aimed to study the relationship between osimertinib exposure and severe toxicity and to identify a safe toxic limit for a preventive dose reduction.

**Methods:**

In this real-life prospective cohort study, patients with NSCLC treated with osimertinib were followed for severe toxicity (grade ⩾3 toxicity, dose reduction or discontinuation, hospital admission, or treatment termination). Blood for pharmacokinetic analyses was withdrawn during every out-patient visit. Primary endpoint was the correlation between osimertinib clearance (exposure) and severe toxicity. Secondary endpoint was the exposure–efficacy relationship, defined as progression-free survival (PFS) and overall survival (OS).

**Results:**

In total, 819 samples from 159 patients were included in the analysis. Multivariate competing risk analysis showed osimertinib clearance (*c.q.* exposure) to be significantly correlated with severe toxicity (hazard ratio 0.93, 95% CI: 0.88–0.99). An relative operating characteristic curve showed the optimal toxic limit to be 259 ng/mL osimertinib. A 50% dose reduction in the high-exposure group, that is 25.8% of the total cohort, would reduce the risk of severe toxicity by 53%. Osimertinib exposure was not associated with PFS nor OS.

**Conclusion:**

Osimertinib exposure is highly correlated with the occurrence of severe toxicity. To optimize tolerability, patients above the toxic limit concentration of 259 ng/mL could benefit from a preventive dose reduction, without fear for diminished effectiveness.

## Background

The most common treatable genetic aberration in patients with non-small cell lung cancer (NSCLC) is a deletion or mutation in the epidermal growth factor receptor (*EGFR*) gene. This oncogenic driver is present in almost 15% of Caucasian patients with non-squamous NSCLC, and even more frequently reported (>40%) in Asian patients.^1,2^ The registration of the first- and second-generation EGFR small-molecule tyrosine kinase inhibitors (SMKIs) markedly increased survival rates compared to conventional chemotherapy in locally advanced and metastatic disease.^3–5^ During treatment with EGFR-SMKIs, an *EGFR* p.T790M resistance point mutation eventually occurs in >60% of patients.^6^ The third-generation EGFR-SMKI osimertinib showed significantly increased progression-free survival (PFS) and overall survival (OS) compared to the other EGFR-SMKIs and proved to be effective against T790M-mutated NSCLC.^7^ These developments have hence caused the median OS of patients with *EGFR*-positive NSCLC to exceed 38 months and the 4-year survival rate to be almost 40%.^8^ Additionally, recent data showed osimertinib to vastly reduce disease recurrence in the adjuvant setting.^9^ As a consequence, many more patients may thus be treated with osimertinib in the future, and also for longer periods of time.

Despite its selectivity for *EGFR*, 20–42% of patients develop grade 3 or higher toxicity, which lead to hospital admissions, treatment discontinuations, and dose reductions.^7–9^ Indirectly, severe toxicity could result in an impaired treatment effect, by interruption or even discontinuation of treatment. These undesirable consequences occurred in up to 25% and 15% of patients, respectively.^7–9^ It is known from a previous population pharmacokinetic (PK) analysis that osimertinib plasma clearance (*c.q.* drug exposure) is correlated with skin rash, diarrhea, and cardiac QTc-time prolongation.^10^ Nevertheless, to date, there are no indicators that can predict severe toxicity beforehand.^11^

Given the importance of osimertinib treatment continuation, in both the metastatic and adjuvant setting, a preventive dose reduction could avoid severe toxicity for patients without impairing treatment effectiveness. Therefore, we performed a prospective cohort study, using samples of patients with NSCLC treated with this agent, to study parameters that influence osimertinib exposure. Herewith, we aimed to study the relationship between drug exposure and occurrence of severe toxicity, and improve osimertinib tolerability by identifying its toxic limit.

## Methods

### Study design and data collection

The START-TKI study^12^ is a real-life, prospective, multi-center cohort study. Patients who are treated with SMKIs at the Erasmus Medical Centre Cancer Institute in Rotterdam and the Amphia Hospital in Breda, both in the Netherlands, between January 2017 and September 2021, were asked to participate in this study. Ethical approval was obtained from the Medical Ethics Committee of the Erasmus Medical Center (MEC 2016-643). Patients treated with osimertinib for locally advanced or metastatic NSCLC according to standard-of-care analyses, who were above the age of 18 years and able to understand and give written informed consent, were selected to be included in this analysis. Since severe toxicity was the primary endpoint of this study, patients were included regardless of disease history, treatment history, T790M- or *EGFR*-mutation, or line of treatment. Patients were only excluded if the treating physician documented possible low or absent treatment adherence. Prior to participation, patients provided written informed consent and were prospectively followed-up until end of osimertinib treatment by their treating pulmonologist. When blood was withdrawn for standard-of-care analyses, an additional blood sample for PK analyses for this study was obtained from all participants. For most patients, this meant that we obtained a PK sample every 3 months. Patients were asked to postpone the intake of osimertinib until the PK sample has been obtained to ensure trough samples. At every visit, osimertinib toxicity was assessed, and a CT scan and laboratory blood analyses (renal function, liver enzymes, and full blood count) were performed. Additionally, patients were asked at what time osimertinib was taken prior to blood withdrawal.

Severe toxicity was defined as toxicity grade ⩾3 scored by the common terminology criteria for adverse events (CTCAE) criteria version 5.0,^13^ or if toxicity led to dose reduction or discontinuation, hospital admission, or termination of osimertinib treatment. The date of hospital admission or dose alteration was used for time-to-event analyses. Additionally, dates of disease progression according to RECIST version 1.1^14^ and death were collected for survival analyses.

Osimertinib plasma concentrations were quantified as described earlier.^15^

### Population PK analysis

PK data were analyzed using nonlinear mixed-effects modeling (NONMEM) version 7.4. Model building was assisted by Perl-speaks-NONMEM version 4.2.0,^16,17^ Pirana software version 2.9.5b,^18^ R version 4.1.1, and Xposed version 4.4.1.^19^

The available data were transformed logarithmically and initially fitted to a one-compartmental linear model. Several model components were tested (i.e. two-compartment PK and different absorption mechanisms) to describe osimertinib PK. Residual error was estimated using an additive error model. Interindividual variability (IIV) in PK parameters was modeled using exponential models. If data below the quantification limit was present and consisted of less than 5% of the data, the M1 method was used.^20^

Continuous covariates were centered on the median and were modeled as power models to explain IIV (see Supplemental Appendix A for all tested covariates). Categorical covariates were modeled as proportional models. Covariate analysis was performed using stepwise forward inclusion (*p* < 0.05) and backwards elimination (*p* < 0.01). Time-varying covariates, such as laboratory parameters, were modeled using the following function:

$$\begin{array}{l} {\mathrm{Lab}}_{\mathrm{current}}={\mathrm{Lab}}_{\mathrm{previous}}+\left({\mathrm{Lab}}_{\mathrm{next}}-{\mathrm{Lab}}_{\mathrm{previous}}\right)\\ \;\;\;\;\;\;\;\;\;\;\;\;\;\;\times \frac{T_{\mathrm{current}}-T_{\mathrm{previous}}}{T_{\mathrm{next}}-T_{\mathrm{previous}}} \end{array}$$

In this equation, Lab is the laboratory value, and *T* stands for time.

The model was evaluated numerically by changes in the objective function value (ΔOFV) and a nonparametric bootstrap procedure (*n* = 30,000). Changes that result in an OFV decrease greater than 3.84, correspond with *p* < 0.05 for one degree of freedom, were considered significant. Changes in the model were evaluated visually using goodness-of-fit plots and visual predictive check plots.

### Exposure–toxicity relationship

After development of the population PK model, differences in median exposure were correlated with severe osimertinib toxicity. Since severe toxicity usually occurs within the first months after treatment initiation,^7^ a cut-off of 12 months was used. Using Cox-regression, univariate time-to-event analyses were performed to identify confounding parameters. Variables with *p* < 0.10 were included in the subsequent multivariate Cox proportional-hazard analysis to correct for bias. Thereafter, the Fine and Gray^21^ competing risk model was performed to ensure the absence of competing risks. For this analysis, a competing risk was defined as cessation of osimertinib therapy as this changed the likelihood of experiencing a toxic event for a patient (e.g. death or change of therapy because of disease progression).

In all the analyses, osimertinib clearance was used as variable for exposure. As all patients started with 80 mg/day, as is clinical practice, IIV was only modeled on clearance; thus, clearance was the best predictor for interindividual differences in exposure. Subsequently, the corresponding trough concentration was calculated to identify the toxic limit in ng/mL.

If osimertinib exposure was significantly correlated with severe toxicity, a toxic limit can be established by using a relative operating characteristic (ROC) curve. In this curve, the optimal sensitivity and specificity of different threshold are visualized. The preventive dose reduction should be effective in decreasing the exposure below the toxic limit, which will be simulated in a large simulation cohort (*n* = 1,000). Thereafter, when osimertinib plasma concentrations were available in the first 2 months of treatment, the trough concentrations were associated with severe toxicity. This was especially done to test the time-to-severe toxicity relationship of the threshold and to confirm its predictive value in clinical practice. Furthermore, in order to assess the risk of toxicity after the dose reduction to 40 mg QD, patients who experienced severe toxicity, and who were dose-reduced, were screened for re-occurrence of severe toxicity.

### Exposure–efficacy relationship

Median osimertinib exposure and PFS and OS were correlated using Cox proportional-hazard univariate analyses. Confounding variables with *p* < 0.10 were used in the Cox proportional-hazard multivariate analyses. If a positive exposure–efficacy relationship exists, a preventive dose reduction should not harm patients by decreasing drug concentrations below normal (*c.q.* effective) levels.

## Results

### Data collection

In total, 819 samples from 159 patients that were obtained between January 2017 and September 2021 were included in the population-PK analysis. A summary of patients’ characteristics is shown in Table 1. One patient suffered from a chronic *Clostridium difficile* infection that hampered osimertinib uptake and was subsequently excluded from the analysis. Median trough level in our population was 226 ng/mL, whereas the median trough level for this patient was 62 ng/mL. Three additional samples were excluded due to non-adherence, as documented in the patient file by the treating physician.

**Table 1.** Patients’ baseline characteristics.

Patient characteristics (*n* = 159)	No. of patients or median	% or IQR
Sex (female)	102	64%
Age (years)	66	60–75
Weight (kg)	69	60–80
Length (cm)	168	162–177
BSA	1.87	1.66–1.99
**Ethnicity**		
Caucasian	140	88%
Southeastern Asian	8	5%
Eastern Asian	7	4%
Western Asian	1	1%
African American	3	2%
**TKI treatment line**		
First-line treatment	66	41%
Second-line treatment	79	50%
Third-line treatment	14	9%
**Prior TKI treatment**		
Erlotinib	56	60%
Afatinib	14	15%
Gefitinib	11	12%
Other	12	13%
**WHO performance score**		
0	32	20%
1	95	60%
2	27	17%
3	5	3%
Primary *EGFR* mutation*		
Classic exon 19 deletion	92	58%
Exon 21 L858R	43	27%
Exon 18 c.2156	5	3%
Rare or compound mutation	19	12%
**Baseline TP53 mutation**		
Yes	85	53%
No	67	42%
Unknown	7	4%
**Follow-up**		
Severe toxicity (months)	9.8	4.6–17.0
Progression free survival (months)	10.2	5.5–18.3
Overall survival (months)	16.6	10.2–25.2
Pharmacokinetic sampling (months)	11.5	5.6–19.4
No. PK samples per patient	3	2–6
No. laboratory samples per patient	9	5–15
**Laboratory values**		
Alkaline phosphatase (U/L)	80	65–110
ALT (U/L)	21	15–30
AST (U/L)	25	21–31
Creatine kinase (U/L)	118	73–189
Gamma glutamyl transpeptidase (U/L)	30	19–54
eGFR (CKD-EPI) (mL/min)	71	59–84
Creatinine (μmol/L)	84	71–97
Hemoglobin (mmol/L)	7.9	7.3–8.6
Hematocrit (L/L)	0.39	0.36–0.42
Thrombocytes (10^9^/L)	213	172–262
Albumin (g/L)	40	37–43
CRP (mg/L)	2.0	0.7–6.3
LDH (U/L)	206	181–241

ALT, alanine aminotransferase; AST, aspartate aminotransferase; BSA, body surface area; CKD-EPI, chronic kidney disease epidemiology collaboration; CRP, C-reactive protein; EGFR, epidermal growth factor receptor; eGFR, estimated glomerular filtration rate; IQR, interquartile range; LDH, lactate dehydrogenase; PK, pharmacokinetics; TKI, tyrosine kinase inhibitor; WHO, World Health Organization.

At data cut-off, severe toxicity occurred in 23% of patients, of which skin toxicity was the most prevalent with 6% occurrence (Table 2). Median time until severe toxicity was 3.7 [interquartile range (IQR) 1.8–6.6 months]. Disease progression according to RECIST occurred in 112 (70%) of patients, and 62 (39%) patients died during the study. Median follow-up is reported in Table 1.

**Table 2.** Incidence of severe osimertinib toxicity in total study cohort.

Specific severe toxicity	*N* = 36 (23%)^	CTCAE gr 1–2	CTCAE gr 3–4	Hospital admission	Dose reduction	Dose termination	Treatment stop
Skin toxicities*	10 (6%)	4	6		9	7	1
CK elevation	7 (4%)	1	6		4	6	
Pneumonitis	5 (3%)	1	5	4	1	3	4
Creatinine increase	4 (3%)	1	3	2	4	4	
AST/ALT increase	3 (2%)	2	1		2	3	
Fatigue	3 (2%)	2	1		2	3	
QTc time prolongation	1 (1%)	.	1		1		
Heart failure	1 (1%)	.	1				1
Diarrhea	1 (1%)	1			1	1	
Thrombocytopenia	1 (1%)	1			1		
Nausea and vomitus	1 (1%)	1			1		
Palpitations	1 (1%)	1			1	1	1

* Rash, paronychia, and acrodermatitis.

^ Two patients experienced two different severe toxicities at the time of dose modification

ALT, alanine aminotransferase; AST, aspartate aminotransferase; CK, creatine kinase; CTCAE, common terminology criteria for adverse events.

### Population PK analysis

A one-compartment model with first-order absorption, first-order elimination, and additive error was best described osimertinib PK (Supplemental Appendix B). Introduction of C-reactive protein (CRP), thrombocyte count, hemoglobin, and alkaline phosphatase as covariates affecting osimertinib clearance improved the model significantly. Other tested covariates did not significantly improve the model (Supplemental Appendix A). The model was particularly improved when adding CRP as a covariate. A 20% increase in exposure is already seen when CRP levels are 20 mg/L. Introduction of all covariates decreased the additive error from 0.221 to 0.176 and decreased the IIV from 33.4% to 27.0%. All evaluations showed that a one-compartment model adequately described the data (Supplemental Appendix C).

### Exposure–toxicity relationship

Osimertinib median clearance in this population was 14.7 (IQR 11.6–18.5) L/h. Osimertinib exposure and age were significantly correlated with severe toxicity in univariate Cox proportional-hazard analysis (both *p* < 0.01) (Supplemental Appendix D). Multivariate competing risks regression analysis showed median osimertinib exposure (HR 0.93, 95% CI 0.88–0.99), and age (HR 1.06, 95% CI 1.02–1.09), to be significantly correlated with severe toxicity. This means that for every liter per hour increase in osimertinib clearance, the risk of severe toxicity is reduced with 7%.

**Figure 1.**
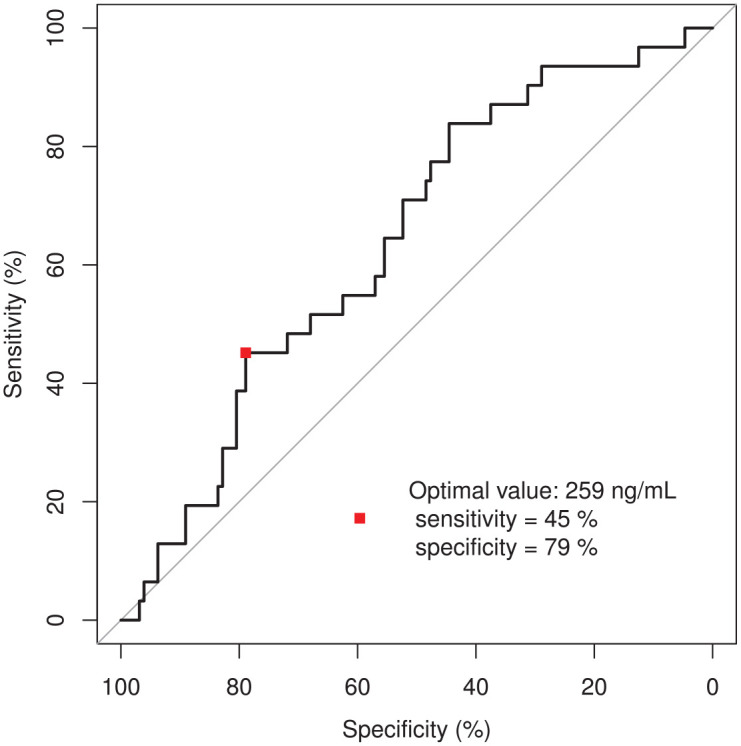
Relative operating characteristic (ROC) curve to determine the optimal osimertinib trough level threshold for toxicity.

**Figure 2.**
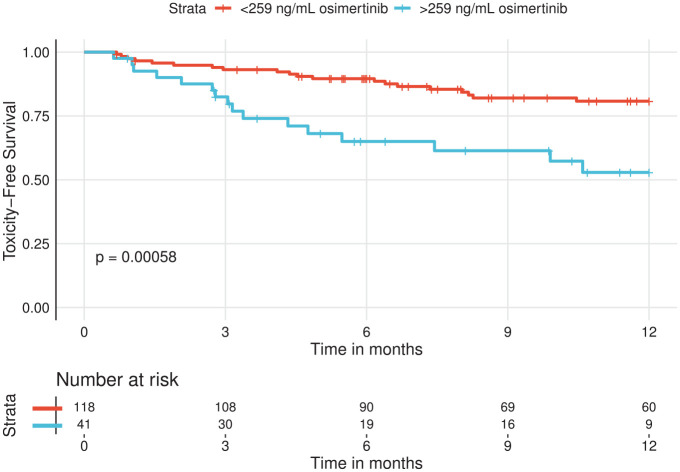
Kaplan–Meier estimates of toxicity-free survival. Patients were stratified as having a higher or lower median osimertinib trough concentration compared to the toxic limit of 259 ng/mL.

When the incidence of severe toxicity and osimertinib exposure was visualized in an ROC curve (Figure 1), the area under the curve was 62.5%. The most sensitive (true-positive) and specific (true-negative) toxic limit would be 259 ng/mL osimertinib. This target concentration divides the cohort into two groups: the risk of severe toxicity in the >259 ng/mL group – 25.8% of the cohort – is 34% *versus* 14% in the <259 ng/mL group. A log-rank test showed the groups to be significantly different (Figure 2). A preventive dose reduction to 40 mg osimertinib QD in the high-exposure group would reduce the risk of severe toxicity by 53%. This is underlined by the finding that from the 21 patients who were dose-reduced to 40 mg QD, only three (14%) experienced re-occurrence of severe osimertinib toxicity.

When stratifying on the occurrence of pneumonitis, which leads to permanent discontinuation of osimertinib treatment, a trend toward increased exposure for patients who experienced pneumonitis was observed (pneumonitis: median plasma concentration [MPC] = 251 ng/mL, standard deviation [SD] = 72 ng/mL; other toxicities: MPC = 241 ng/mL, SD = 85 ng/mL; no toxicities: MPC = 214 ng/mL, SD = 92 ng/mL). Due to the small number of patients who experienced a pneumonitis, this difference was nonsignificant (*p* = 0.25).

In the study cohort, osimertinib concentrations in the first 2 months after start of treatment were available for 90 patients. After this time period, most events of severe toxicity started to occur (Figure 2). Correlation of the first plasma trough concentrations in this time period revealed a similar difference in severe toxicity of almost 50% (31% *versus* 17%), when dividing the cohort into two by the toxic limit of 259 ng/mL osimertinib (Supplemental Appendix E).

**Figure 3.**
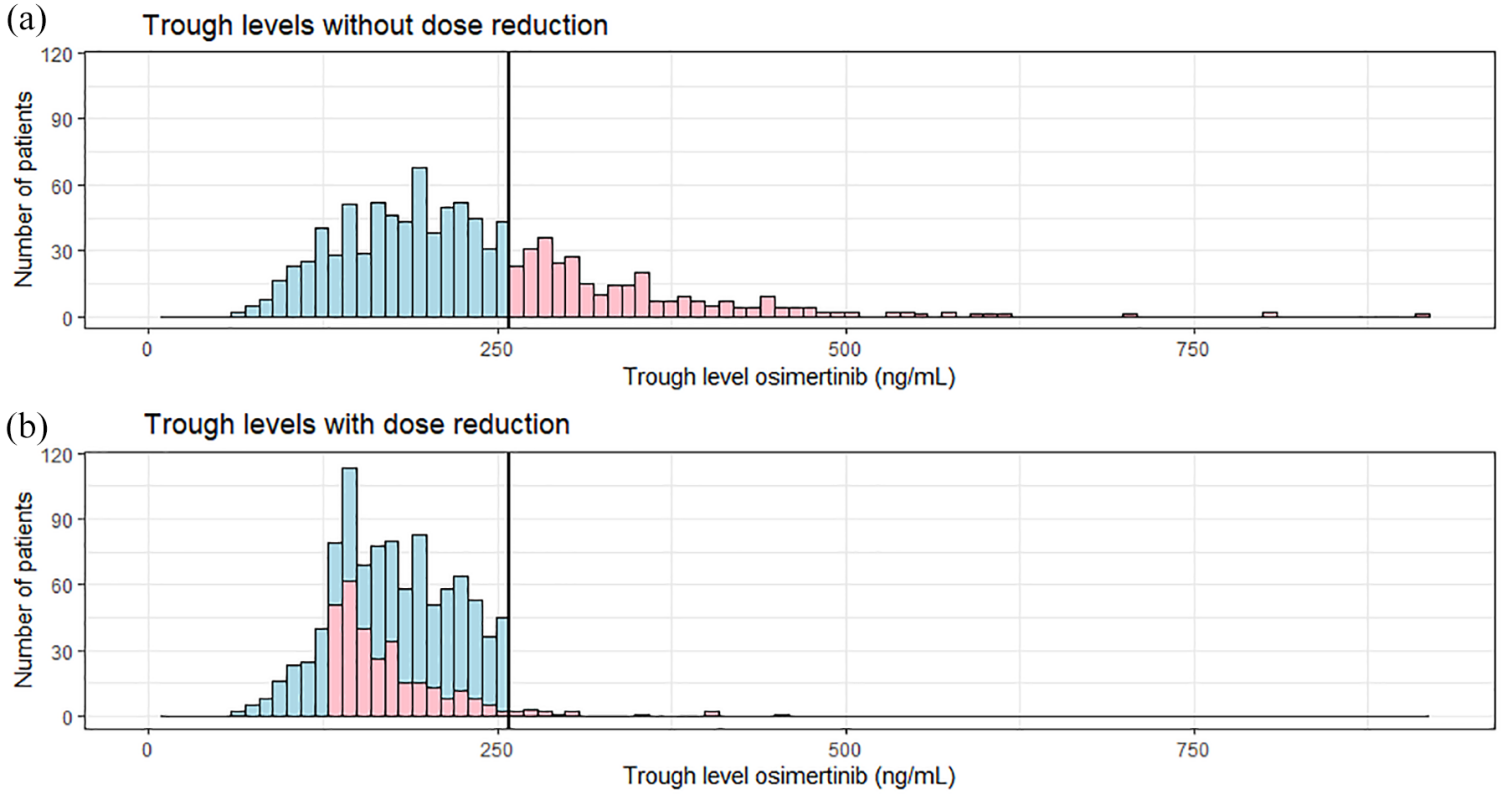
Dose reduction effectively lowers osimertinib trough levels. (a) Distribution of osimertinib trough levels in a simulation cohort consisting of 1000 patients. The proposed toxic limit is visualized as a black vertical line (259 ng/mL). (b) Simulated distribution if the proposed 50% dose-reduction is applied for patients who were above the toxic limit in part (a).

When the osimertinib exposure was simulated after the proposed 50% dose reduction, the range in exposure was similar to the exposure in the patients without a dose reduction (median trough levels: 173.1 *versus* 180.1 ng/mL, and SDs: 45.3 *versus* 46.3 ng/mL) (Figure 3).

### Exposure–efficacy relationship

Osimertinib exposure was significantly and negatively correlated with PFS in univariate Cox regression (*p* = 0.04) (Supplemental Appendix D). After correction for median CRP, median alkaline phosphatase, sex, age, *EGFR* mutation type, and TP53 mutations, the effect became non-significant (HR 0.95, 95% CI 0.91–1.00; *p* = 0.05). For OS, a similar correlation was observed in univariate Cox regression (*p* < 0.01). After correction for CRP, alkaline phosphatase, hemoglobin, primary *EGFR* mutation, and WHO performance status >1, only a trend toward significance remained for osimertinib exposure (HR 0.95, 95% CI 0.89–1.00; *p* = 0.10).

## Discussion

This is the first study that describes osimertinib exposure to be significantly correlated with the occurrence of severe toxicity, and to suggest a safe, preventive dose reduction based on a toxic limit concentration of 259 ng/mL osimertinib.

Our data are supported by a prior study that also found a correlation with any grade toxicity.^10^ The proposed toxic limit of 259 ng/mL osimertinib from our real-life study could result in a 53% reduction in severe toxicity for 26% of patients. This could prevent treatment discontinuation and subsequent treatment failure. Of course, in real life, other environmental factors may still influence the exposure to the drug (e.g. drug–drug and food–drug interactions),^22,23^ which might therefore result in other toxicity outcomes, and the findings in this study should therefore be prospectively validated.

Importantly, we did not find a significant multivariate correlation between median osimertinib exposure and survival. The initial univariate–inverse relationship between exposure and survival was confounded by known parameters that are associated with cachexia (CRP, alkaline phosphatase, and hemoglobin)^24^ and important baseline characteristics (primary *EGFR* mutation and WHO performance status).^25,26^ These results are in line with a prior osimertinib PK model study that reported an absent exposure–efficacy relationship over the 20–240 mg dose range.^10^ A dose reduction of 50% would thus be safe, but should be validated prospectively.

The toxic limit is based on the median exposure during the total treatment time. When only samples are used prior to the occurrence of the majority of severe toxicity (*c.q.* before 2 months after treatment initiation), a similar effect occurred. This underlines the predictability and clinical implementability of our results. Since osimertinib reaches a steady-state concentration after 14 days of treatment, we suggest to perform osimertinib quantification after 14 days to forestall early toxicity.

The principle of a toxicity-preventing dose reduction based on therapeutic drug monitoring (TDM) is very common and frequently applied in daily clinical practice, for example, in the field of infectious diseases^27^ and transplantation medicine.^28^ In the field of medical oncology, a preventing dose reduction based on TDM is less common. Most anticancer drugs, SMKIs in particular, are flat-dosed at the maximum tolerated dose and are only dose reduced after severe toxicity occurs.^29^ Whereas, ideally, this should be done beforehand to avoid toxicity. For example, chemotherapeutic agents are sometimes individually dosed on expected exposure, which is predicted on individual patient characteristics (e.g. *DPYD* polymorphisms, body weight, estimated glomerular filtration rate [eGFR], and length), as is the case for capecitabine and carboplatin.^30,31^ For pemetrexed^32^ and taxanes,^33^ exposure–toxicity relationships have been studied and also here, dose adjustments have been proposed to further optimize the treatment of individual patients.

Osimertinib drug costs of 80 and 40 mg QD in the Netherlands are exactly the same, currently both €6.150 per patient per month.^34^ It would, hence, be financially interesting to consider dosing patients, eligible for a toxicity-preventing dose reduction, 80 mg every other day instead of 40 mg QD. This would potentially save 13% of total osimertinib drug costs. Since osimertinib has a long half-life of more than 40 h,^35^ this would be pharmacologically feasible.

The validity of our population PK model is indirectly confirmed by the similarity with a previously published model.^10^ In our model, especially CRP proved to be a strong, clinically relevant biomarker to predict osimertinib exposure. This is not surprising, since inflammation causes downregulation of CYP450 enzymes and subsequently affects the PK of various other drugs.^36^ This finding could further lead to a temporary dose reduction when patients suffer from inflammation. Since quantification of osimertinib is not routine practice for most hospitals, a faster and simple CRP test would be more feasible to include in routine laboratory checks and should be validated prospectively.

A limitation of our study was an absent *a priori* power analysis, which causes the statistical analyses to be of a retrospective nature. However, the chance of a statistical type II error of these results is relatively small, because of the relatively large size of this cohort. A second limitation could be the different covariates that influence osimertinib exposure that complicate clinical interpretation. Nevertheless, despite the smaller group of 90 patients with samples during the first 2 months, the uncorrected values from these months predicted severe toxicity as well. This confirms that clinical extrapolation is definitely warranted.

To conclude, osimertinib exposure is significantly correlated with the occurrence of severe toxicity. Tolerability of osimertinib could, if prospectively validated, be optimized by implementation of a safe, preventive dose reduction in patients above the toxic limit of 259 ng/mL

## Supplemental Material

sj-docx-1-tam-10.1177_17588359221103212 – Supplemental material for Improving the tolerability of osimertinib by identifying its toxic limit Click here for additional data file. Supplemental material, sj-docx-1-tam-10.1177_17588359221103212 for Improving the tolerability of osimertinib by identifying its toxic limit by Bram C. Agema, G. D. Marijn Veerman, Christi M. J. Steendam, Daan A. C. Lanser, Tim Preijers, Cor van der Leest, Birgit C. P. Koch, Anne-Marie C. Dingemans, Ron H. J. Mathijssen and Stijn L. W. Koolen in Therapeutic Advances in Medical Oncology
