# Supplementary material for: Improving the tolerability of osimertinib by identifying its toxic limit
Source: Ther Adv Med Oncol. 2022 Jun 3;14:17588359221103212. doi: 10.1177/17588359221103212 (PMC9168866; doi:10.1177/17588359221103212)
Supplement: sj-docx-1-tam-10.1177_17588359221103212 – Supplemental material for Improving the tolerability of osimertinib by identifying its toxic limit [file sj-docx-1-tam-10.1177_17588359221103212.docx]

**SUPPLEMENTARY MATERIALS**

**APPENDIX A**

**COVARIATE EQUATIONS**

**Power model:**

$$P_{i}={CL}_{pop}* \left( \frac{COV}{median COV} \right)^{\Theta_{COV}}*{exp}^{\left( \eta_{i} \right)}$$

**Exponential model:**

$$P_{i}={CL}_{pop}* \left( \frac{COV}{median COV} \right)*\Theta_{COV}*{exp}^{\left( \eta_{i} \right)}$$

**Categorical model**

$$P_{i}={CL}_{pop}* {\Theta_{Cat}}^{FLAG}*{exp}^{(\eta_{i} )}$$

Tested covariates on clearance:

- Demographics
  - Age
  - Weight
  - BMI
  - BSA
  - Sex
  - Ethnicity
  - WHO performance status
- Laboratory liver/kidney parameters
  - Alkaline phosphatase
  - ALAT
  - ASAT
  - Albumin
  - Creatine kinase
  - Gamma-glutamyl transferase
  - Estimated glomerular filtration rate
  - Creatinine
- Complete blood count
  - Haemoglobin
  - Haematocrit
  - Thrombocyte count
- Other
  - Lactate dehydrogenase
  - C-reactive protein

**APPENDIX B: MODEL BUILDING AND DIAGNOSTICS**

**Model building**

**At first, a 1-compartmental model with first-order absorption was fitted to the log transformed data. A 2-compartmental model let to a numerically better fit according to OFV but to a poorer fit according to the AIC. Additionally, the 2-compartment model did not visually improve the fit and was therefore not incorporated in the model. As the estimated absorption rate deviated from prior reported values and the RSE was large, we tested multiple mechanistic absorption models, lag time, zero-order absorption, fixing it on the reported values, adding IIV, and adding transit compartments. This did not lead to improvement of the model and therefore the deviation of the absorption rate constant was accepted. A one-compartment model with first-order absorption, first order elimination and a proportional error was subsequently used for the covariate analysis.**

**Introduction of CRP as a covariate decreased the OFV by 254 points and reduced the error from 0.221 to 0.187. It also stabilized and decreased absorption rate constant and explained 6% of the IIV. Thereafter, the introduction of thrombocytes (dOFV=-63), haemoglobin (dOFV=-28), and alkaline phosphatase (dOFV=-25) proved to be a significant improvement and were incorporated in the final model. Initially, LDH (dOFV=-4.6) and sex (dOFV=-4.6) also improved the model significantly. However, LDH and sex were excluded from the model** after the more stringent backward elimination (p < 0.01). Other covariates such as albumin did not significantly improve the model after addition of CRP, thrombocytes, haemoglobin and alkaline phosphatase. CRP, thrombocytes, haemoglobin and alkaline phosphatase were incorporated in the final model. The covariates decreased the proportional error from 0.221 to 0.176 and decreased the IIV from 33.4% to 27%. **Model estimates and bootstrap results are shown in table A1.**

**Table B1:** Parameter estimations and bootstrap results of the final osimertinib model

| Parameter (unit) | Parameter estimate [shrinkage] | | RSE (%) | | Bootstrap Median | | 95% CI bootstrap |
| --- | --- | --- | --- | --- | --- | --- | --- |
| Ka (h^-1^) | 0.332 | | 30.7 | | 0.349 | | 0.20 – 0.77 |
| V/F (L) | 1150 | | 7.4 | | 1154 | | 1019 – 1372 |
| CL/F (h^-1^) | 14.50 | | 2.4 | | 14.59 | | 13.9 – 15.3 |
| Covariates on CL/F | | | | | | | |
| CRP | -0.119 | | 11.4 | | -0.118 | | -0.15 - -0.09 |
| Thrombocytes | -0.317 | | 20.3 | | -0.319 | | -0.44 - -0.19 |
| Haemoglobin | 0.576 | | 26.2 | | 0.585 | | 0.30 – 0.89 |
| ALK-P | -0.155 | | 25.0 | | -0.150 | | -0.23 - -0.07 |
| IIV |  | |  | |  | |  |
| CL (CV%) | 26.8 [6.3] | | 12.3 | | 26.8 | | 23.2 – 30.5 |
| Residual error | |  | |  | |  | |
| *Proportional (%)* | 17.50 [8.9] | | 3.2 | | 17.42 | | 16.3 – 18.6 |
| Conditional number | 5.09 | |  | |  | |  |

Abbreviations: Ka =absorption constant; V/F = distribution volume divided by bioavailability; CL = drug clearance; CRP = c-reactive protein; ALK-P = alkaline phosphatase; IIV = inter-individual variance.

**APPENDIX C MODEL DIAGNOSTICS**

Figure C1: Goodness of fit plots for the final osimertinib model. Abbreviations: IWRES: individual weighted residuals


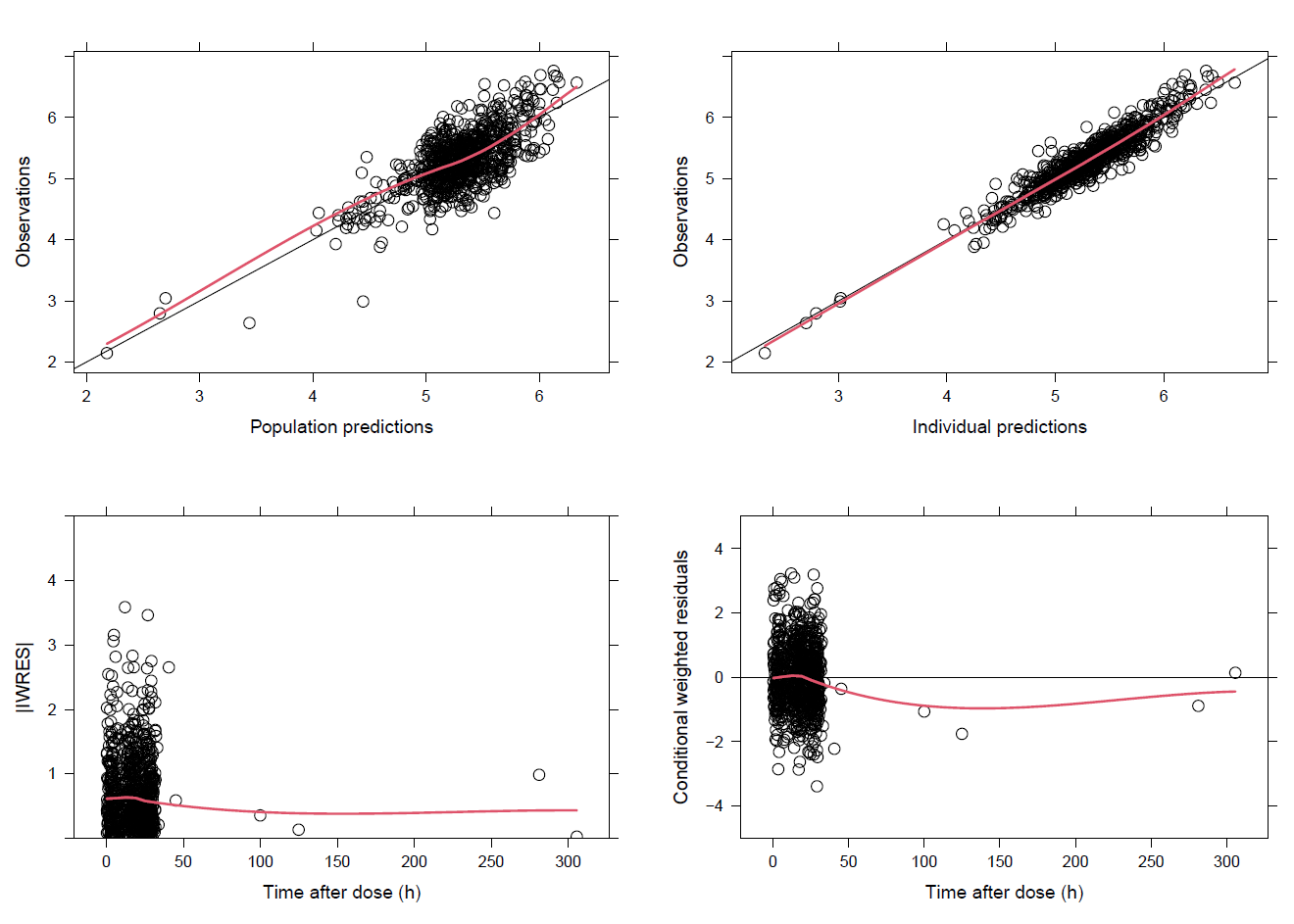


Figure C2: Visual Predictive Checks (VPC’s) for covariates included in the final model.


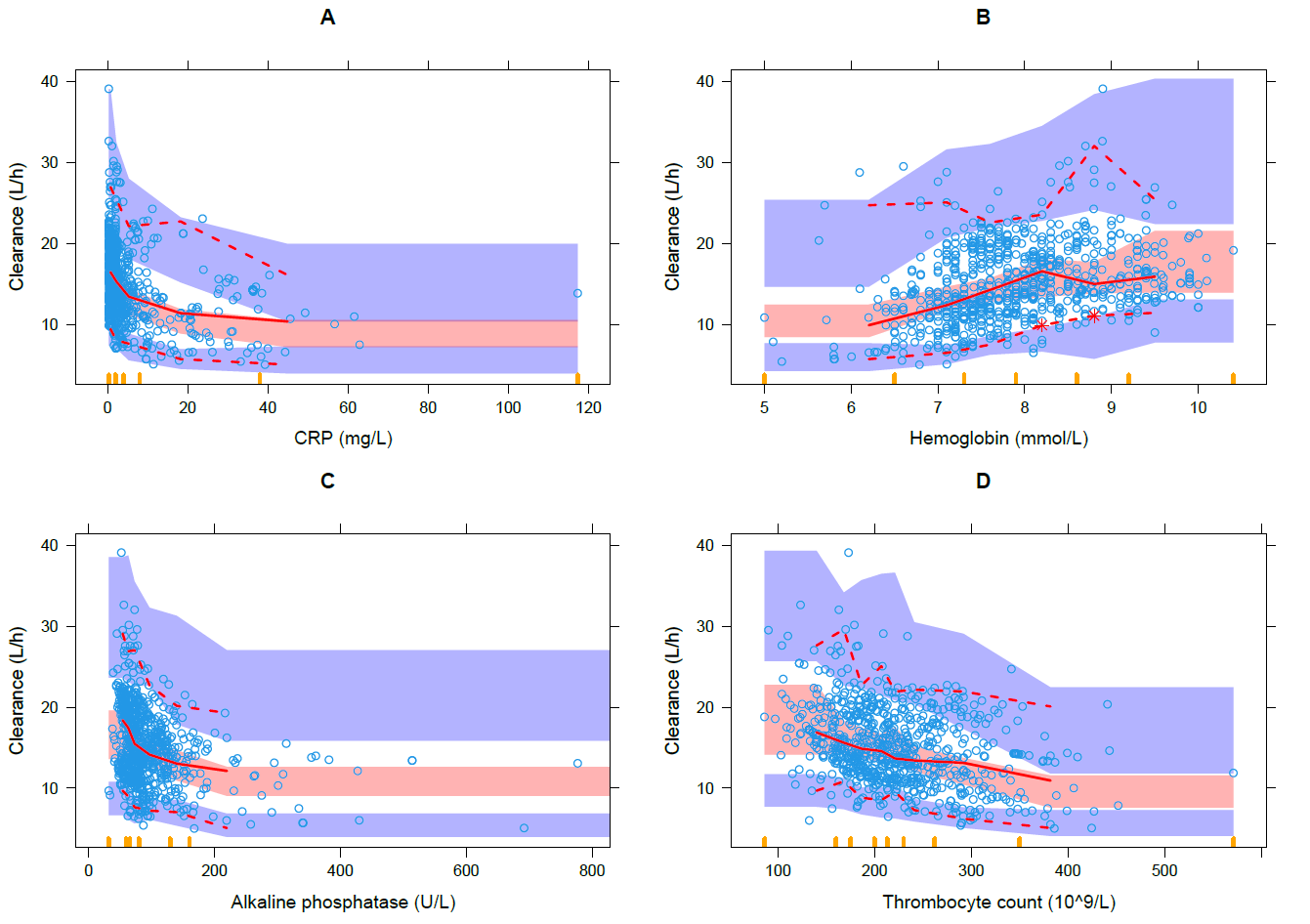


**APPENDIX D OUTCOMES OF COX PROPORTIONAL-HAZARD MODEL ANALYSES.**

Table S1: Outcomes of univariate Cox proportional-hazards models for severe toxicity.

| Factor | HR | 95% CI | Grouping |
| --- | --- | --- | --- |
| Clearance | 0.92 † | 0.85 - 0.99 | Median per person (L/h) |
| CRP | 0.98 | 0.92 - 1.05 | Median per person (mg/L) |
| ALKP | 1.00 | 1.00 - 1.01 | Median per person (U/L) |
| Hemoglobin | 0.80 | 0.50 - 1.27 | Median per person (mmol/L) |
| Thrombocytes | 1.00 | 1.00 - 1.00 | Median per person (*10^9/L) |
| Sex | 1.16 | 0.55 - 2.46 | Male vs. Female |
| Age at start | 1.05 † | 1.01 - 1.08 | (Years) |
| EGFR mutation | 0.65 | 0.34 - 1.26 | Exon 19 del vs. others |
|  | 1.29 | 0.64 - 2.63 | L858R vs. others |
|  | 1.47 | 0.64 - 3.35 | Compound/rare vs. other |
| TP53 mutation | 0.92 | 0.46 - 1.84 | No mutation vs. mutation |
| TKI-line | 0.60 | 0.30 - 1.22 | First line vs. Second/third line |
| Metastases in CNS | 1.02 | 0.37 - 2.82 | No CNS metastases vs. CNS metastases |
| WHO PS | 1.60 | 0.71 - 3.58 | WHO 0&1 vs. WHO 2&3 |

* = p<0.10

† = p<0.05

‡ = p<0.01

Table S2: Outcome of multivariate Cox proportional-hazards model for severe toxicity

| Factor | HR | 95% CI | Grouping |
| --- | --- | --- | --- |
| Clearance | 0.90 ‡ | 0.84 - 0.97 | Median per person (L/h) |
| Age at start | 1.06 ‡ | 1.02 - 1.10 | (Years) |

* = p<0.10

† = p<0.05

‡ = p<0.01

Table S3: Outcomes of univariate Cox proportional-hazards models for progression-free survival.

| Factor | HR | 95% CI | Grouping |
| --- | --- | --- | --- |
| Clearance | 0.95 † | 0.91 - 1.00 | Median per person (L/h) |
| CRP | 1.05 † | 1.03 - 1.05 | Median per person (mg/L) |
| ALKP | 1.00 † | 1.00 - 1.01 | Median per person (U/L) |
| Hemoglobin | 0.98 | 0.78 - 1.24 | Median per person (mmol/L) |
| Thrombocytes | 1.00 | 1.00 - 1.01 | Median per person (*10^9/L) |
| Sex | 0.67 † | 0.46 - 0.99 | Male vs. Female |
| Age at start | 0.98 * | 0.97 - 1.00 | (Years) |
| EGFR mutation | 0.66 † | 0.45 - 0.96 | Exon 19 del vs. others |
|  | 1.36 | 0.91 - 2.04 | L858R vs. others |
|  | 1.41 | 0.82 - 2.40 | Compound/rare vs. other |
| TP53 mutation | 1.77 † | 1.22 - 2.60 | No mutation vs. mutation |
| TKI-line | 1.10 | 0.85 - 1.44 | First line vs. Second/third line |
| Metastases in CNS | 0.80 | 0.47 - 1.36 | No CNS metastases vs. CNS metastases |
| WHO PS | 1.34 | 0.84 - 2.15 | WHO 0&1 vs. WHO 2&3 |

* = p<0.10

† = p<0.05

‡ = p<0.01

Table S4: Outcome of multivariate Cox proportional-hazards model for progression-free survival

| Factor | HR | 95% CI | Grouping |
| --- | --- | --- | --- |
| Clearance | 0.95* | 0.91 - 1.00 | Median per person (L/h) |
| CRP | 1.03‡ | 1.01 - 1.06 | Median per person (mg/L) |
| ALKP | 1.00 | 1.00 - 1.00 | Median per person (U/L) |
| Sex | 0.64 † | 0.41 - 0.99 | Male vs. Female |
| Age at start | 0.99 | 0.96 - 1.00 | (Years) |
| EGFR mutation | 0.65 † | 0.44 - 0.97 | Exon 19 del vs. others |
| TP53 mutation | 1.60 † | 0.63 - 1.06 | No mutation vs. mutation |

* = p<0.10

† = p<0.05

‡ = p<0.01

Table S5: Outcomes of univariate Cox proportional-hazards models for overall survival.

| Factor | HR | 95% CI | Grouping |
| --- | --- | --- | --- |
| Clearance | 0.90‡ | 0.85 - 0.96 | Median per person (L/h) |
| CRP | 1.05‡ | 1.03 - 1.08 | Median per person (mg/L) |
| ALKP | 1.01‡ | 1.00 - 1.01 | Median per person (U/L) |
| Hemoglobin | 0.68† | 0.50 - 0.93 | Median per person (mmol/L) |
| Thrombocytes | 1.00 | 1.00 - 1.01 | Median per person (*10^9/L) |
| Sex | 0.86 | 0.52 - 1.45 | Male vs. Female |
| Age at start | 0.99 | 0.97 - 1.02 | (Years) |
| EGFR mutation | 0.53† | 0.32 - 0.87 | Exon 19 del vs. others |
|  | 1.31 | 0.76 - 2.28 | L858R vs. others |
|  | 2.08† | 1.14 - 3.77 | Compound/rare vs. other |
| TP53 mutation | 1.01 | 0.66 – 1.54 | No mutation vs. mutation |
| TKI-line | 1.15 | 0.67 – 1.93 | First line vs. Second/third line |
| Metastases in CNS | 1.13 | 0.82 – 1.56 | No CNS metastases vs. CNS metastases |
| WHO PS | 1.85‡ | 1.24 – 2.77 | WHO 0&1 vs. WHO 2&3 |

* = p<0.10

† = p<0.05

‡ = p<0.01

Table S6: Outcome of multivariate Cox proportional-hazards model for overall survival

| Factor | HR | 95% CI | Grouping |
| --- | --- | --- | --- |
| Clearance | 0.95 | 0.89 - 1.01 | Median per person (L/h) |
| CRP | 1.04‡ | 1.01 - 1.08 | Median per person (mg/L) |
| ALKP | 1.00† | 1.00 - 1.00 | Median per person (U/L) |
| Hemoglobin | 0.80 | 0.57 - 1.13 | Median per person (mmol/L) |
| EGFR mutation | 0.50† | 0.27 - 0.91 | Exon 19 del vs. others |
|  | 1.20 | 0.59 - 2.45 | Compound/rare vs. others |
| WHO PS | 3.06‡ | 1.35 - 4.40 | WHO 0&1 vs. WHO 2&3 |

* = p<0.10

† = p<0.05

‡ = p<0.01

**APPENDIX E SUBANALYSIS FIRST OSIMERTINIB PLASMA CONCENTRATION**

Figure D1: First osimertinib plasma through concentration plotted median osimertinib plasma trough concentration. The vertical line is the toxic limit of 259 ng/mL, the diagonal line is the unity line, red dots are patients that experienced severe toxicity in the first 12 months of treatment, and the grey see-through pane is the 30% variability interval.

**
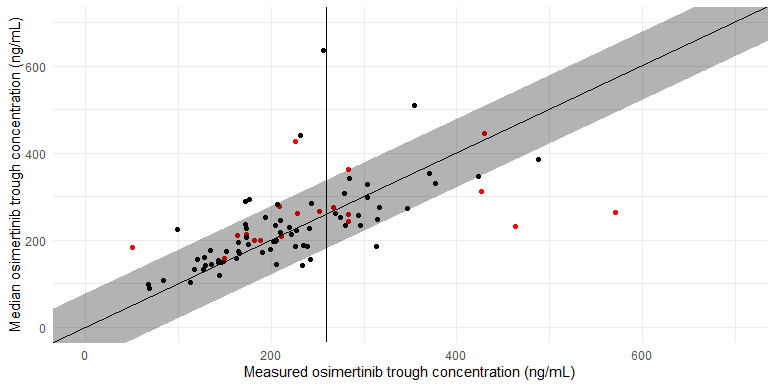
**

**APPENDIX F FINAL MODEL CONTROL STREAM**

$INPUT CENSOR ID DATE=DROP TIME DVLN DV DOSE=AMT ADDL II TAD CMT EVID MDV SEX AGE LENGTH WEIGHT WHO ALKP ALKPPRE DATEALKPPRE ALKPPOST DATEALKPPOST ALAT ALATPRE DATEALATPRE ALATPOST DATEALATPOST ALB ALBPRE DATEALBPRE ALBPOST DATEALBPOST ASAT ASATPRE DATEASATPRE ASATPOST DATEASATPOST CK CKPRE DATECKPRE CKPOST DATECKPOST EGFR EGFRPRE DATEEGFRPRE EGFRPOST DATEEGFRPOST CRP CRPPRE DATECRPPRE CRPPOST DATECRPPOST

GGT GGTPRE DATEGGTPRE GGTPOST DATEGGTPOST HEMOGLOB HEMOGLOBPRE DATEHEMOGLOBPRE HEMOGLOBPOST DATEHEMOGLOBPOST HEMATOCR HEMATOCRPRE DATEHEMATOCRPRE HEMATOCRPOST DATEHEMATOCRPOST KREAT KREATPRE DATEKREATPRE KREATPOST DATEKREATPOST LDH LDHPRE DATELDHPRE LDHPOST DATELDHPOST THROMBO THROMBOPRE DATETHROMBOPRE THROMBOPOST DATETHROMBOPOST ETHNIC BASEWEIGHT LASTDOSE CLMED ROWNUM TIMEAE LASTTIME

$DATA Dataset.csv IGNORE=C;

$SUBROUTINES ADVAN2 TRANS2

$PK

CALLFL=-2

MTDIFF=1

MTIME(1) = DATEALKPPRE

MTIME(2) = DATEALKPPOST

ALKPDIFF = ALKPPOST-ALKPPRE

ALKPTIME = TIME-MTIME(1)

IF(ALKPTIME.LE.0) ALKPTIME=0.001

ALKPINT = MTIME(2)-MTIME(1)

IALKP = ALKPPRE+(ALKPDIFF*(ALKPTIME/ALKPINT))

MTIME(1) = DATECRPPRE

MTIME(2) = DATECRPPOST

CRPDIFF = CRPPOST-CRPPRE

CRPTIME = TIME-MTIME(1)

IF(CRPTIME.LE.0) CRPTIME=0.001

CRPINT = MTIME(2)-MTIME(1)

ICRP = CRPPRE+(CRPDIFF*(CRPTIME/CRPINT))

MTIME(1) = DATEHEMOGLOBPRE

MTIME(2) = DATEHEMOGLOBPOST

HEMOGLOBDIFF = HEMOGLOBPOST-HEMOGLOBPRE

HEMOGLOBTIME = TIME-MTIME(1)

IF(HEMOGLOBTIME.LE.0) HEMOGLOBTIME=0.001

HEMOGLOBINT = MTIME(2)-MTIME(1)

IHEMOGLOB = HEMOGLOBPRE+(HEMOGLOBDIFF*(HEMOGLOBTIME/HEMOGLOBINT))

MTIME(1) = DATETHROMBOPRE

MTIME(2) = DATETHROMBOPOST

THROMBODIFF = THROMBOPOST-THROMBOPRE

THROMBOTIME = TIME-MTIME(1)

IF(THROMBOTIME.LE.0) THROMBOTIME=0.001

THROMBOINT = MTIME(2)-MTIME(1)

ITHROMBO = THROMBOPRE+(THROMBODIFF*(THROMBOTIME/THROMBOINT))

KA = THETA(2)

TVCL = THETA(3) * ((ICRP/2.1)**THETA(5)) * ((ITHROMBO/214)**THETA(6)) * ((IHEMOGLOB/7.8)**THETA(7)) * ((IALKP/81)**THETA(8))

CL = TVCL * EXP(ETA(1))

V = THETA(4)

S2 = V/1000 ;scaling from ng/mL to mg/L

$THETA

(0, 0.24) ;1 prop err

(0, 0.0084) ;2 Ka

(0, 14.2) ;3 CL

(0, 1986) ;4 V

(-10, 1) ;5 CRP

(-10, 1) ;6 THROMBO

(-10, 1) ;7 HEMOGLOB

(-10, 1) ;8 ALKP

$ERROR ;; Calculation based on log-transformed data

IPRED=LOG(0.0001)

IF(F.GT.0)IPRED=LOG(F)

W=1

IF(F.GT.0)W = SQRT(THETA(1)**2)

IRES = DV-IPRED

IWRES = IRES/W

Y = IPRED+W*EPS(1)

$OMEGA

(0.46) ; IIV CL

$SIGMA

1 FIX ; Proportional error PK

$EST METHOD=1 INTER MAXEVAL=2000 NOABORT SIG=3 PRINT=1 POSTHOC

$COV PRINT=E
